# Clinical Potential of Immunotherapies in Subarachnoid Hemorrhage Treatment: Mechanistic Dissection of Innate and Adaptive Immune Responses

**DOI:** 10.14336/AD.2023.0126

**Published:** 2023-10-01

**Authors:** Anke Zhang, Yibo Liu, Xiaoyu Wang, Houshi Xu, Chaoyou Fang, Ling Yuan, KaiKai Wang, Jingwei Zheng, Yangjian Qi, Sheng Chen, Jianmin Zhang, Anwen Shao

**Affiliations:** ^1^Department of Neurosurgery, The Second Affiliated Hospital, School of Medicine, Zhejiang University, China.; ^2^Key Laboratory of Precise Treatment and Clinical Translational Research of Neurological Diseases, Hangzhou, Zhejiang, China.; ^3^Clinical Research Center for Neurological Diseases of Zhejiang Province, Hangzhou, China.; ^4^Department of Neurosurgery, Shanghai General Hospital, School of Medicine, Shanghai Jiao Tong University, Shanghai, China.

**Keywords:** subarachnoid hemorrhage, innate immunity, adaptive immunity, immunotherapy

## Abstract

Subarachnoid hemorrhage (SAH), classified as a medical emergency, is a devastating and severe subtype of stroke. SAH induces an immune response, which further triggers brain injury; however, the underlying mechanisms need to be further elucidated. The current research is predominantly focused on the production of specific subtypes of immune cells, especially innate immune cells, post-SAH onset. Increasing evidence suggests the critical role of immune responses in SAH pathophysiology; however, studies on the role and clinical significance of adaptive immunity post-SAH are limited. In this present study, we briefly review the mechanistic dissection of innate and adaptive immune responses post-SAH. Additionally, we summarized the experimental studies and clinical trials of immunotherapies for SAH treatment, which may form the basis for the development of improved therapeutic approaches for the clinical management of SAH in the future.

Subarachnoid hemorrhage (SAH) is a devastating subtype of stroke, occurring at a mean age of 55 years. It causes both vascular and neural pathologies and leads to poor neurological outcomes [1, 2]. Although the incidence rate of SAH is lower than that of ischemic stroke or intracerebral hemorrhage, accounting for only 5% of strokes, its high incidence in the young population and high mortality makes the death toll similar to that of the more common subtypes of stroke [3, 4]. Innate and adaptive immunity represents the predominant branches of the immune responses that are critical for the initiation and development of inflammation post-SAH. Innate immunity is the first line of defense against harmful stimuli, while adaptive immunity is a long-term process that retains the memory of antigen exposure; however, these two types of immunities are closely interrelated. At present, studies primarily focus on the role of innate immune cells post-SAH, while the information on adaptive immunity and potential immunotherapies post-SAH is limited.

In this review, we primarily focused on the immune responses post-SAH onset and the impact of cerebral and systemic immunity during SAH progression; additionally, we summarized the various immunotherapeutic strategies used for SAH treatment.

## SAH pathophysiology and immune response

The pathophysiological processes involved in SAH are complex and have not yet been determined [5]. Some pathophysiological changes post-SAH, such as neuroinflammation, vasospasm, cerebral edema, and delayed cerebral ischemia (DCI), affect the prognosis of SAH patients [6, 7]. SAH is primarily caused by aneurysms, and early brain injury (EBI), which usually occurs within the first 3 d post-SAH onset, has been recognized as a major factor contributing to the poor prognosis of SAH patients [8, 9]. Post-SAH EBI is a multifactorial process with a predominantly primary injury. The post-SAH hemorrhage causes a sudden increase in intracranial pressure, forcing the brain tissue into a state of ischemia and hypoxia through a series of processes that may be important initiating factors for EBI. A large amount of blood enters the subarachnoid space immediately after SAH and accumulates in the cerebral sulcus and cerebral pools to form a hematoma that compresses the brain tissue, resulting in several pathological processes, including increased intracranial pressure, decreased cerebral blood flow and cerebral perfusion pressure, and cerebral edema [10]. The development of cerebral edema and hydrocephalus may further increase intracranial pressure, thus exacerbating EBI development [11]. The hemodynamic changes post-SAH, such as ischemia and hypoperfusion of the brain tissues, cellular hypotonicity caused by the disruption of antidiuretic hormone secretion, and the destruction of the basement membrane structure of the blood-brain barrier (BBB), lead to the development of brain edema [12-15].

In some patients, cerebral vasospasm, the constriction of the large intracranial arteries that occurs 3 to 14 d post-SAH, and inadequate cerebral perfusion due to cerebral vasospasm were thought to be the primary causes for DCI post-SAH, making the prevention and treatment of cerebral vasospasm an important modality in SAH management [16]. Some pathological processes, such as vascular smooth muscle injuries, inflammatory responses, and vascular wall remodeling, can lead to impaired diastolic function of the cerebral vasculature, further leading to vasospasm [16].

Immune responses post-SAH onset are involved in the subsequent development of EBI, vasospasm, cerebral edema, and DCI. Neuroinflammation mediated by microglia and other immune cells plays a key role in EBI development. In animal studies, it was observed that microglia were dynamically polarized from M1 to M2 phenotype and significantly aggregated at the injury site within 1 to 10 d post-SAH [17]. Due to the destruction of erythrocytes after hemorrhage, excess free heme can stimulate microglia to polarize to the M1 phenotype and promote neuroinflammation. Some blood degradation products, such as damage-associated molecular patterns (DAMPs), can bind to pattern recognition receptors (PRRs) on the immune cells, such as microglia, and activate immune responses; therefore, removal of these degradation products reduces the SAH-induced activation of inflammatory reactions [18-20]. Neutrophils are thought to be the first peripheral immune cells to enter the central nervous system (CNS) post-SAH [21], and inactivation of neutrophils may reduce the development of neuroinflammation and thus EBI [22]. In addition, neurovascular coupling dysfunction post-SAH leads to microvascular dysfunction, which can exacerbate EBI [23]. In addition, post-hemorrhagic neuroinflammation and oxidative stress damage to brain structures at the BBB can cause and exacerbate EBI [24]. Moreover, neuroinflammatory damage to cerebrovascular endothelium and smooth muscle cells can lead to vasodilatory dysfunction and consequently vasoconstriction spasm, which is an important factor in DCI [24].

## Innate and adaptive immunity

**Figure 1.**
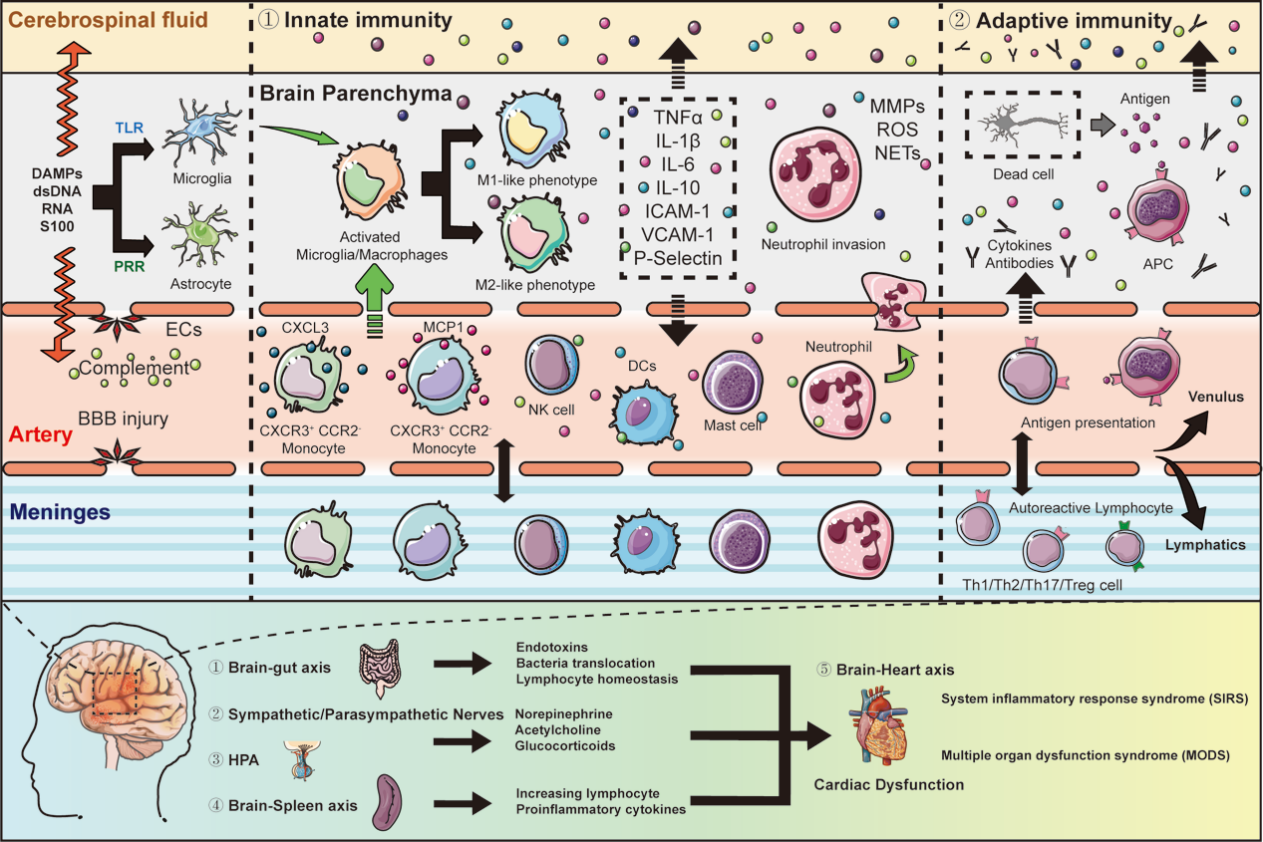
Alteration of cerebral and systemic immunity after SAH onset. SAH onset damages cells and release DAMPs and dsDNA. Then DAMPs initiate innate immunity by secretion of cytokines, and in turn, triggers neutrophil invasion. Additionally, circulating immune cells, especially neutrophil, adhere to the endothelium and traffick into the brain parenchyma and meninges. Invaded neutrophils further injure the brain cells with ROS, MMPs, cytokines, and neutrophil extracellular traps (NETs). Initiation of the complement cascade is another pathway of cell damage. In terms of adaptive immunity, dead cells release antigens and contact with APCs in the brain. APCs, in turn, engage naive lymphocytes for further chronic inflammation and cytotoxicity. Damage triggers activation of the HPA axis and nervous system, leading to secretion of glucocorticoids and catecholamines. Brain-derived DAMPs leak into the peripheral circulation and activate systemic immunity, mobilizing innate immune cells form lymphoid organs, spleen, and gut, which further leads to manifestation of SIRS, like cardiac dysfunction.

Innate immune cells include neutrophils, microglia, macrophages, mast cells (MCs), natural killer (NK) cells, dendritic cells (DCs), as well as specific types of lymphocytes. Immune recognition activates innate immune cells and a variety of effector responses [25-27]. The dominant effector response is pathogen elimination by phagocytosis and secretion of cytokines and pro-inflammatory mediators, such as tumor necrosis factor-α (TNF-α), interleukin (IL)-6, and reactive oxygen species (ROS), which directly eliminate potential threats via a massive and undifferentiated inflammatory response [28]. In contrast, adaptive immunity that consists of cell- and humoral-mediated immunity (via T and B lymphocytes, respectively) conveys antigen-specific immune responses based on high-affinity receptors, and several studies have reported the essential role of T lymphocytes in SAH. Therefore, SAH engages both innate and adaptive immune systems, which play an essential role in the progression of brain damage. The alteration of cerebral and systemic immunity post-SAH onset is shown in Figure 1.

## SAH and innate immunity: acute immune responses

Innate immune cells in the circulatory system are rapidly engaged at the onset of aneurysm rupture, causing a high infiltration of blood-borne immune cells in the brain parenchyma, thus activating brain-resident immune cells.

### BBB disruption and inflammation

SAH onset disrupts BBB integrity, exposing the brain parenchyma to neurotoxic substances and immune cells, ultimately triggering secondary brain injuries [29]. The sudden disruption of an aneurysm and rapidly rising intracranial pressure post-SAH decelerates cerebral blood flow (CBF) and induced global cerebral ischemia, along with hemocyte extravasation, endothelial alteration, and platelet-leukocyte adhesion. These pathological alterations can also lead to the contraction of endothelial cells (ECs), the disintegration of tight junctions, and a further increase in vascular permeability [30, 31]. Additionally, increased BBB permeability results in the transmigration of immune cells, especially leukocytes, into the microenvironment. Both leukocytes and DAMPs can activate the toll-like receptor (TLR)-4 on microglia and initiate inflammatory cascades [32, 33]. Additionally, binding to TLR4 facilitates the production of pro-inflammatory cytokines, such as TNF-α, IL-1β, IL-6, IL-8, IL-12, and matrix metalloproteases (MMP)-9, which consequently triggers abnormal infiltration of macrophages and neutrophils into the brain parenchyma.

### Complement system

The complement system, which may be a major component of humoral immunity during acute SAH, consists of opsonins (iC3b, C3dg, and C3d) and anaphylatoxins (C3a and C5a). Anaphylatoxins are dominant pro-inflammatory mediators that induce vasoconstriction and coagulation and promote the production and release of cytokines, degranulation, and phagocytosis via complement receptors on myeloid cells. These processes play an essential role in the occurrence of EBI and DCI. Moreover, the plasma levels of mannose-binding lectin, C3a, and C5a post-SAH are associated with the prognosis of SAH patients [34, 35]. C5a contributes to the progression of EBI post-SAH, and deletion of C5a or inactivation of C5a via C5-specific antibodies reduces neuroinflammation and alleviates brain injury [36]. The initiators of the lectin complement pathway initiate complement cascades post-SAH and may contribute to severe brain injuries and poor prognosis of SAH patients [35, 37, 38].

### Brain-resident immune cells

#### Parenchymal microglia

Microglia are the predominant brain-resident immune cells conducting antigen presentation, phagocytosis, and cytokine production post-SAH [39]. The early neuroinflammation reaction in the first 48 h after SAH onset is predominantly initiated by the resident microglia rather that the infiltrated monocytes [40]. Although BBB disruption facilitates macrophage infiltration, the majority of immune cells activated by day 5 post-SAH onset are Ccr2^-^/Cx3cr1^+^ microglia rather than Ccr2^+^/Cx3cr1^+^ macrophages, indicating that the accumulating Iba1^+^ cells are predominantly derived from the resident microglia [17]. A wide range of transcription factors and cytokines activate microglia by altering their morphology in response to various brain insults. Two distinct forms of microglia orchestrate neuroinflammation post-SAH. The M1-like phenotype drives the pro-inflammatory responses to tissue injuries, while the M2-like microglia are involved in anti-inflammation, angiogenesis, and tissue repair [41, 42]. During acute SAH, microglia are activated by the stimuli from the extravasate blood and are primarily of M1 phenotype with a ramified morphology [43]. Increasing evidence also revealed the critical role of microglia in EBI post-SAH. During acute SAH, microglia present morphological alteration and increase in the regions adjacent to the blood clot [44], after which they gradually transform to the M2-like phenotype, thus suggesting their role in natural restoration and protection in the delayed SAH phase. This delayed transformation might also contribute to the occurrence of DCI and secondary brain damage post-SAH.

#### Meningeal and perivascular macrophages

The role of the other brain-resident immune cells, including meningeal and perivascular macrophages, in SAH, is not entirely known. Previous studies have revealed that parenchymal microglia mediate the clearance of erythrocytes in the subarachnoid space [45-47]; however, due to the limited spatial contribution of blood around the brain, it is unclear how microglia are exposed to the erythrocytes. Moreover, the depletion of perivascular and meningeal macrophages exerts neuroprotection within 24 h post-SAH onset [17]. Furthermore, based on the anatomical location, perivascular and meningeal macrophages may be the first to interact with erythrocytes and be involved in the initiation of immune responses in brain parenchyma post-SAH.

#### MCs

MCs form a sizable population in several brain regions, including meninges, choroid plexus, mesencephalon, hippocampus, and entorhinal cortex [48-50] and predominantly reside in the perivascular region. MCs are in contact with vessel cells, neurons, and microglia and contribute to acute neurobehavior and subsequent cognition [51, 52]. They contain granules with vasoactive agents and trigger the accumulation of pro-inflammatory cytokines, causing further brain injuries post-SAH, which can be alleviated by histamine and thromboxane inhibitors [53-55].

### Peripheral immune cells

#### Neutrophils

Neutrophils infiltrate the injured site during the acute SAH phase and reach a peak within 48 h post-SAH [56]. Increased neutrophil levels and inflammation are observed in the peripheral immune system post-SAH [57, 58]. Neutrophils contribute to the aggravation of inflammatory responses, brain edema, hypoperfusion, and neuronal damage via the neutrophil-endothelial interaction and release of proteases (such as MMP-9), ROS, and IL-1β [59-62]. Additionally, neutrophils promote coagulation and further exacerbate microcirculation disorders [63]. Therefore, targeting neutrophils may be a potential strategy to inhibit neutrophil-endothelial interactions and attenuate cerebral hypoperfusion through targeting neutrophils [64].

#### Myeloid cells

A previous study found increasing infiltration of neutrophils, followed by increasing infiltration of classical monocytes in the brain within 2 d post-SAH. These results indicate increased monocyte traffic in the brain parenchyma and increased monocyte differentiation towards the M1-like phenotype in the acute SAH phase after aneurysm rupture [57]. Monocyte-derived macrophage (MDM) infiltration of the brain parenchyma requires CCL2 signaling, and suppression of MDM recruitment improves SAH prognosis [65, 66]. It has been demonstrated that PD-1^+^/Ly6c^+^/CCR2^+^ monocytes originate from the bone marrow (BM) following carotid artery perforation, in response to catecholamines, and mediate cerebral vasospasm post-SAH [67]. Brain-resident microglia react within 24 h post-SAH, while MDM can be observed up to 72 h in the brain parenchyma [68]. In general, the distinct functional MDMs can be classified into M1- and M2-like phenotypes. M1-like macrophages are characterized by high expression of CD68 and CCR7 antigens [69], while M2-like macrophages are characterized by high expression of CD163 and CD206 antigens [70]. Similar to microglia, pro-inflammatory M1-like macrophages are associated with acute inflammation, while anti-inflammatory M2-like macrophages are associated with tissue repair and regeneration [71]. In addition, the CD68^+^ macrophages conduct phagocytosis for the clearance of cellular debris [72]. Previous studies demonstrated that targeting the CD11b/CD18 integrin receptor or deleting MDMs prior to SAH induction can reduce the occurrence of cerebral vasospasm [44, 73].

#### DCs

DCs are antigen-presenting cells with the ability to activate and regulate cell differentiation of naïve T cells. DCs express various TLRs and mediate the activation of adaptive immunity. Furthermore, DCs release IL-17 and IL-23, which can further drive the polarization of TH17 cells [74]. A previous study found a significant decrease in both myeloid and plasmacytoid DCs in SAH patients, which may be associated with the inhibited DC differentiation of BM progenitors [75-77]. Additionally, owing to the early pathogen detection ability of DCs, decreasing DC levels in the circulatory system may cause immunosuppression, which explains the high incidence rate of pneumonia post-SAH.

#### NK cells

NK cells are highly specialized effector cells that mediate immune responses via cytotoxicity and cytokine production [78, 79]. Cytotoxic NK cells induce vessel narrowing of cerebral arteries and contribute to DCI during acute SAH [80]. A clinical study observed increased activated cytotoxic NK cells in the cerebrospinal fluid (CSF) post-SAH and increased risk of CV and DCI, suggesting the involvement of NK cells in the pathogenesis of cerebrovascular complications in SAH [81]. Cytotoxic NK cells express high levels of integrins, including lymphocyte function-associated antigen-I (LFA-I) and macrophage antigen-I (Mac-I), allowing their entry from circulation via ECs and into inflammatory sites and mediating endothelial damages [82, 83]. Notably, blocking LFA-I or Mac-I using monoclonal antibodies can prevent both vascular inflammation and CV [73, 84]. Therefore, the transmigration of NK cells across the vascular wall mediates EC lysis and contributes to the dysfunction of cerebral arterial vessels in SAH patients.

## SAH and adaptive immunity: chronic inflammation

Adaptive immunity is mediated by a variety of T cell receptors (TCRs) present on the surface of T lymphocytes, which recognize pathogens and monitor the host immune response [85, 86]. Activated lymphocytes facilitate both the neutralization of microbes and the clearance of damaged cells by secreting various cytokines [87, 88]. Several studies have also confirmed the role of T lymphocyte-mediated immunity in peripheral circulation post-SAH [89, 90]. Additionally, it has been revealed that lymphocytes are involved in the pathophysiology of SAH based on the lymphocytic infiltration in the arteries by cerebral vasospasm [91]. A previous study confirmed that T lymphocytic infiltration in the subarachnoid space is closely associated with the cerebral blood vessels in the SAH model [91]. T cell-derived IL-1β was also detected in CSF from the SAH patients, which indicates the misregulation of T lymphocytes in the microenvironment of SAH manifestation [92]. Moreover, CD4^+^ T lymphocyte (T helper cells) infiltration is high and reaches peak levels in the early SAH models. Peripheral T cells are significantly increased on day 3 post-SAH [91]. Moreover, aggressive co-stimulation profiles and elevated T helper cell levels are observed in SAH patients with cerebral vasospasm [93]. Over the past decades, several subsets of CD4^+^ T cells, including Th1, Th2, Th17, and T-regulatory (Treg) cells, have been identified and reported to display dynamic changes and exert effects during EBI post-SAH [94-97]. According to an *in vivo* study, the administration of stains enhances the shift from Th1 cells to Th2 cells in the SAH model [98]. Furthermore, a significant increase in Th17 cells and a decrease in Th2 cells was observed post-SAH, in a clinical study [90]. Furthermore, the proliferative capacity of Treg cells, as suppressor lymphocytes, is impaired post-SAH [99], which is associated with the increased adhesion of T lymphocytes and co-stimulatory properties of the Treg cells in the peripheral circulation of SAH patients [99]. In addition, FOXP3^+^ Treg cells release a series of immunosuppressive cytokines, including IL-10, TGF-β, and IL-35, which inhibit the activity of immune cells and increase immune tolerance post-SAH [100, 101]. Altogether, these results suggest the indispensable role of CD4^+^ T cells post-SAH onset.

## Entry points of immune cells post SAH

### BBB

The BBB is an indispensable unit of the neurovascular system that mediates substance communication between the peripheral circulation and CNS [102]. BBB dysfunction is characterized by the degradation basement membrane and the loss of ECs, pericytes, and astrocytes within a few hours post-SAH [103]. BBB disruption occurs within 30 mins and injury peaks at 72 h post-SAH onset [104]. Furthermore, the disruption of BBB or the CSF drainage system can be considered a communication channel for immune cells [105, 106]. EC damage causes the release of cell adhesion molecules (CAMs), which further induce infiltration of immune cells, including neutrophils and macrophages, from peripheral circulation and cause inflammation post-SAH [107].

### Meninges and Choroid plexus

Although immune cells are unable to enter the brain parenchyma under homeostasis, the meninges are populated by several immune cells, including T cells, DCs, MCs, and macrophages, which provide immune surveillance and regulate brain functions [108]. Furthermore, owing to the connection between vascular channels and dura mater, monocytes and neutrophils may enter the brain parenchyma via this alternative route [109, 110]. Meningeal lymphatic vessels, which are typical lymphatic structures that mediate drainage of molecular contents and lymphocytes to the peripheral circulation, have been observed and investigated in the meninges [111, 112]. SAH onset impairs the polarization of astrocyte aquaporin-4 (AQP4), a biomarker of glymphatic clearance, leading to the accumulation of various T lymphocytes in the brain parenchyma [113]. Although the role of meningeal immunity post-SAH is not clearly understood, its constitution and maintenance are critical aspects of tissue immunology.

The choroid plexus (CP) serves as another entry point for the circulating lymphocytes, especially CD4^+^ T cells [114, 115]. Due to the fenestrated endothelium, the CP inflammatory responses and CP are also linked. Infiltration of the circulating immune cells leads to increased levels of CCL2, TNF-α, and ILs in the CP post-SAH [116]. Moreover, SAH induces the infiltration of epi-plexus macrophages with CD68, CD163, CCR7, CD206, and MHC immunophenotypes and promotes cell proliferation of MDMs in the CP [117]. In addition, the CP also serves as a site for the infiltration of leukocytes, which present both beneficial and detrimental effects. The influx of peripheral leukocytes, especially neutrophils, into the brain parenchyma during acute SAH may deteriorate hemorrhagic brain injuries [118]. To promote leukocyte trafficking, the CP epithelium constitutively expresses ICAM-1 and VCAM-1[119], as well as the chemoattractant molecule, CC chemokine ligand 20 (CCL20), which binds to CCR6 on the surface of TH17 cells to enter the CSF [120].

### Skull BM

A recent study reported that the skull BM may serve as the origin of meningeal immune cells. It demonstrated that the skull BM-derived neutrophils, rather than those derived from the tibial-BM, are easier to traffic to the adjacent brain parenchyma in stroke and aseptic meningitis models. A study on the skull-dura interface revealed that myeloid cells migrate across the inner skull cortex, suggesting a direct interaction between the brain and the skull BM [110]. Furthermore, a few studies reported the presence of monocytes and neutrophils in the meninges, which were supplied from the adjacent skull and vertebral BM rather than peripheral circulation. Resident myeloid cells derived from the skull BM are distinct from their blood-derived counterparts in transcriptional signatures. Myeloid cells in the adjacent BM niches are more likely to supply innate immune cells under various conditions [121]. However, the role of skull BM-derived immune cells post-SAH is poorly understood and the entry of immune cells into the brain parenchyma during brain injury requires further studies to determine their therapeutic potential.

### SAH and systemic immunity

Systemic immunity is involved in the acute SAH phase, and systemic inflammatory responses are activated post-SAH and carry an increased risk of CV, DCI, and systemic complications [122-124]. Approximately 50% of SAH patients present systemic inflammatory responses, while 85% of SAH patients present systemic inflammatory response syndrome (SIRS) within the first 4 d post-SAH [125]. Following brain injury, DAMPs and cytokines enter the circulatory system and exacerbate systemic immune responses. Brain injuries further activate neurohumoral pathway, which contribute to immune activation. Brain-derived DAMPs and cytokines produced during acute phase after SAH onset leak into the circulation through disrupted BBB or CSF drainage of meningeal lymphatic vessels [126].

### Circulatory system

Following brain injury DAMPs and cytokines enter the circulatory system and exacerbate systemic immune responses. Brain injuries further activate the neurohumoral pathway, which contributes to immune activation. Brain-derived DAMPs and cytokines produced during acute SAH leak into the circulation through disrupted BBB or CSF drainage of meningeal lymphatic vessels [126]. A few studies demonstrated that circulating high mobility group box-1 (HMGB-1) increases during acute SAH and blocking HMGB-1 using monoclonal antibodies confers protective functions [127, 128]. Some experimental studies observed elevated serum cytokines (MMP-9, IL-1β, and IL-6) and increased inflammatory mediators (TNFα, IL-6, IL-2, and CCL2) post-SAH [129-131]. Similar results were observed in SAH patients, where MMP-9 and IL-6 levels were elevated post-SAH, and the serum levels of MMP-9 and IL-6 were associated with the severity and poor prognosis of SAH [132]. Additionally, serum levels of IL-23 and IL-17 increased in SAH patients and were associated with post-hemorrhagic complications [133].

### Autonomic nervous system

SAH-induced damage may cause autonomic nervous dysfunction, which may further induce the release of inflammatory cytokines. The autonomic nervous system regulates the secretion of catecholamines and elevated sympathetic tone due to brain damage can cause an increase in catecholamine release [134], which may lead to subendocardial hemorrhages or contraction band necrosis in the heart or pulmonary edema in the lungs [135].

### Sympathetic nervous system and hypothalamic-pituitary-adrenal (HPA) axis

The activation of peripheral innate immunity post-SAH is also regulated and controlled by the sympathetic nervous system (SNS) and the HPA axis, resulting in the release of norepinephrine, acetylcholine, and glucocorticoids, and further manifesting of SIRS [136-138]. SIRS is characterized by the alteration of body temperature, heart rate, blood pressure, and leukocytosis and usually results in subsequent tissue hypoperfusion [139, 140]. Furthermore, activated systemic inflammation may provoke innate immune responses in the CNS and deteriorate BBB destruction [141]. Activation of the SNS and HPA axis is considered the first mode of communication between the CNS and the peripheral immunity post-SAH onset [142, 143]. Moreover, activation of the SNS can also increase the Treg cell population, thereby presenting immunosuppressive function. The pro-inflammatory cytokines released by the damaged cells trigger the posterior hypothalamus to augment sympathetic output and secrete norepinephrine, acetylcholine, and glucocorticoids [144, 145]. According to the data obtained from SAH patients, plasma catecholamine and cortisol levels are elevated post-SAH onset [146-148].

### Brain-gut axis

The brain-gut axis can influence brain function in healthy as well as pathological conditions, such as acute brain injury (ABI) [149-151]. The brain-gut axis, composed of neural and humoral pathways, can regulate immune response and lymphocytic expansion [152]. ABI-induced gut dysbiosis can further affects neuroinflammatory and immune responses in the CNS and determine neurological function [153].

Stress-mediated gut dysbiosis can regulate T cell homeostasis, enhancing T lymphatic migration from the intestine to the brain and promoting pro-inflammatory responses, ultimately leading to unexpected clinical outcomes in ischemic stroke [152]. SAH patients are frequently accompanied by various gastrointestinal dysfunctions, including gastrointestinal bleeding, gastric reflux, and decreased intestinal peristalsis, which further exacerbate SAH prognosis [154]. The initiation of a cascade of intestinal dysfunction events, including over-production of intestinal cytokines, increased intestinal permeability and accelerated bacterial and endotoxin entry in the intestines [155], affect the function and integrity of remote organs, resulting in SIRS and multiple organ dysfunction syndrome. The dominant alterations of SAH-induced gastrointestinal dysfunction include stress ulcer formation, gut motility dysfunction, and gut barrier disruption [154, 156]; however, gut dysbiosis-mediated immune responses post-SAH have not yet been fully investigated.

### Brain-spleen axis

Spleen serves as an essential organ in the regulation of the peripheral immune response, owing to the elevated levels of circulating lymphocytes and pro-inflammatory cytokines, including TNF-α, interferon-γ (IFN-γ), IL-6, and IL-2 and increased inflammation during the acute phase after stroke [157]. Additionally, the spleen plays a critical role in regulating systemic immunity after stroke and serves as the dominant source of brain MDMs [158-160]; however, the brain-spleen interaction post-SAH is not yet fully understood. It has been revealed that a total of 98% of splenic innervation consists of sympathetic nerve fibers [161]. Therefore, these mediators can induce splenic atrophy, T cell apoptosis, and NK cell deficiency. Furthermore, the activation of SNS and HPA post-SAH can lead to splenic contraction; however, studies focusing on the interactions between the spleen-brain axis are limited.

### Brain-heart axis

Systemic immunity may arise due to SAH-induced brain injuries and cardiac damage. It has been demonstrated that catecholaminergic stress coincides with immune cell infiltration in the heart and leads to further damage post-SAH [105]. Infiltrated neutrophils and thrombi can be observed in the inflamed myocardium. Parasympathetic dysfunction and the release of catecholamines can regulate inflammatory response and trigger myocardial dysfunction and cardiomyocyte apoptosis [105]. According to an investigation of SAH patients, increasing levels of catecholamines is associated with the prolongation of QT-interval and myocardial damage post-SAH [162, 163]. In addition, activation of the SNS in the early SAH period exacerbates myocardial damage and contributes to cardiac dysfunction. Thus, intensive heart monitoring is particularly necessary for SAH patients [164]. Furthermore, increased gut permeability allows the leakage of bacteria and endotoxins into the circulatory system, triggering the infiltration of pro-inflammatory cytokines, which further exacerbates cardiac dysfunction [165, 166].

**Figure 2.**
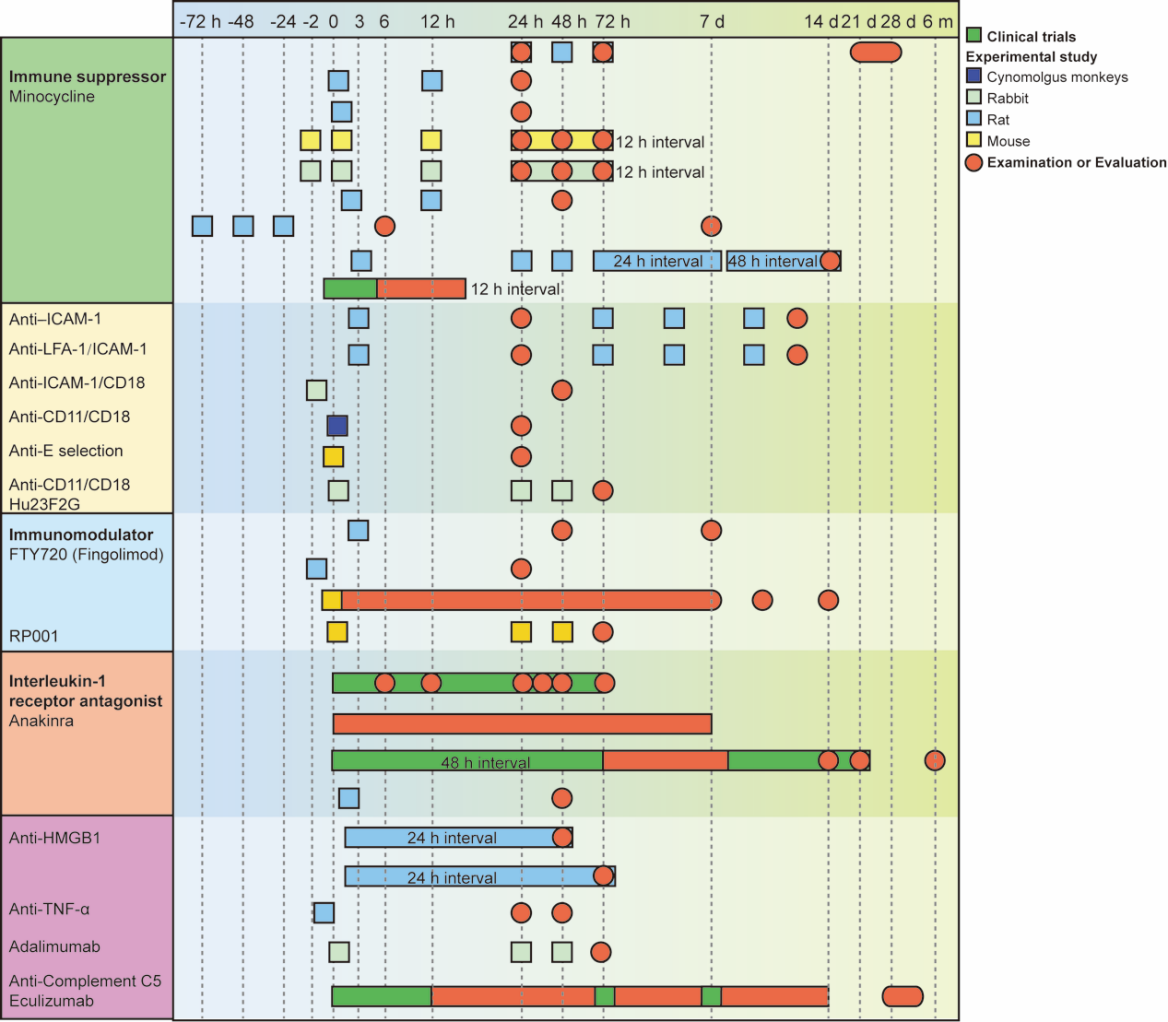
Time course of selected immunomodulatory therapies for SAH. For each immunomodulatory therapy, the experimental studies and clinical trials are listed in chronological order, with the length of the bars indicating the treatment period and observative window. Clinical trials and experimental studies in different animal models were distinguished by colors.

## Post-SAH immunity as a therapeutic target

Immunotherapy for SAH is predominantly focused on inhibiting immune responses and improving prognosis. Previous studies have confirmed that immune-related markers serve as diagnostic or prognostic markers in SAH patients [167, 168]. The neutrophil-to-lymphocyte ratio (NLR) in patients with aneurysmal SAH (aSAH) has been shown to act as a prognostic marker. In a meta-analysis, it was shown that NLR, as an inflammatory index, independently predicted poor functional outcomes in SAH and was associated with the occurrence of DCI [167]. Additionally, blood leukocyte and neutrophil levels were found to be associated with vasospasm. Higher leukocyte and neutrophil counts were detected in the blood of patients with vasospasm within 2 weeks post-SAH [169]. Therefore, therapeutic modalities that target these immune cells and related markers are promising in SAH treatment.

Currently, several potential immunomodulatory drugs may be used for SAH treatment. Inflammatory factor-mediated (e.g., IL-1 and IL-6) inflammation is an important factor in DCI occurrence post-SAH; therefore, antagonists targeting inflammatory factors can effectively improve SAH prognosis by reducing neuroinflammation. Activated immune cells (e.g., microglia and lymphocytes) release inflammatory factors; therefore, inhibitors targeting immune cells can reduce the release of inflammatory factors by inhibiting immune cell activation. Ultimately, the reduction of neuro-inflammation can reduce neural cell death, reduce cerebral edema, and protect vascular structures, thus providing cerebral protection post-SAH. In addition, targeting CAMs and circulating factors can also inhibit the development of neuroinflammation. In this section, we summarized the experimental studies and clinical trials using immunomodulatory therapies for SAH treatment. The time course, dose selection, injection approach, therapeutic window, and functional outcomes of all the studies have been reviewed in Figure 2 and Tables 1 and 2.

**Table 1:** Review of human trials using agents targeting the immune system in SAH patients.

Drug	Trial type	Drug vs placebo	Test items	Age	Fisher scales	Therapeutic window	Dose and injection method	Outcomes	Year	Ref.
Interleukin-1 receptor antagonist Anakinra	Phase I	6 patients	CSF and plasma samples were taken with 72 h.	39-69	3-4	within 5 days of SAH	i.v. bolus injection 100 mg	IL-1RA reached a steady-state plasma concentration of 2264 lg/mL by 15 mins, and CSF concentrations were maintained at 78 to 558 ng/mL between 1 and 24 h.	2008	[191]
Interleukin-1 receptor antagonist Anakinra	Pharmacokinetic modelling	8 patients	A full physical examination was performed on days 0, 1, 2, 3, 4, 10 and 30. blood biochemistry and CSF were monitored at these time points.	39-69	2-3	within 5 days of hemorrhage	i.v. bolus injection 100 mg	Peripherally administered IL-1RA crosses slowly into and out of the CSF of patients with SAH.	2008	[194]
Interleukin-1 receptor antagonist Anakinra	Two-center, randomized, double-blind, controlled trial.	6 vs 7	CSF and plasma samples were taken at 6, 12, 24 (end of infusion), 36, 48, and 72 hours.	40-69	3-4	within 72 hours from ictus	i.v. bolus injection 500 mg	Reduction of IL-6 concentration in both CSF and plasma for SAH patients treated with IL-1Ra.	2014	[193]
Interleukin-1 receptor antagonist Anakinra	open-labelled design. Phase II	25 vs 4	CSF and plasma samples were taken with 7 days.	37-76	NA	4 h infusion	i.v. bolus (100-1000 mg) and 4 h (1-10 mg/kg/h)	A dosage regimen of 500 mg intravenous bolus and 10 mg/kg/h could achieve the target concentration in CSF	2016	[236]
Interleukin-1 receptor antagonist Anakinra	Three-center, open-label, randomized controlled Phase II trial.	68 vs 68	Blood sampling daily from Days 3 to 8, and then on Days 14 and 21 post-ictus. Glasgow Outcome Scale-extended score at 6 months.	23-77	1-4	twice daily until a maximum of 21 days	Sc. injection	Anakinra is safe and well-tolerated and can effectively suppresses the IL-1-mediated response in SAH patients.	2018	Galea [196]
Complement C5 Antibody Eculizumab	A randomized, controlled, open-label, phase II clinical trial	13 vs 13	CSF sample and neurological examination during 1-14 days, and functional outcomes in 4, 10, and 13 weeks.	> 18	NA	< 12 h, on day 3 and day 7 after ictus	i.v. bolus injection (1200 mg)	Evaluating pharmacodynamic efficacy and safety of eculizumab in the early phase after aSAH.	2020	[217]
Immune suppressor Minocycline	A double-blinded randomized trial.	6 vs 5	Peripheral serum samples were drawn at day 0 to day 14 or discharge. Vasospasm was evaluated at day 3-14	44-68	3-4	24-hour increments for a total of 4 days	i.v. bolus injection 10 mg/kg (maximum 700 mg daily)	Minocycline curtails breakdown of the BBB following aSAH as evidenced by lower permeability indices.	2021	[206]

### Immunomodulator-fingolimod

Fingolimod (FTY720) is a high-affinity agonist of sphingosine-1-phosphate receptors (S1PRs) that can prevent lymphocytes against egress from lymph nodes and entry into the peripheral circulation, and then resulting in lymphopenia and decreased lymphocytic inflammation [170, 171]. In addition to its immunosuppressive effects on lymphocyte trafficking, FTY720 can also cross the BBB and directly modulate S1PR-positive cells, especially microglia [172-175]. Moreover, several studies have revealed the neuroprotective effects of FTY720 in CNS injury models. Previous experimental studies and clinical trials proposed that FTY720 can alleviate secondary brain damage and present therapeutic efficacy in patients with ischemic stroke and intracranial hemorrhage (ICH) [176-178]. In SAH, FTY720 has been observed to attenuate neuronal cell death during EBI and suppress inflammation by decreasing TNF-α, IL-6, and IL-8 levels, thus alleviating neurological function and inhibiting brain edema [179, 180]. Moreover, FTY720 significantly increased Treg cells, decreased NK cells, and limited the intravascular adhesion of leukocytes to pial venules to attenuate brain damage and facilitate neurological recovery post-SAH [181, 182]. Notably, RP001, a novel analog of FTY720 with greater affinity for S1PR1 and S1PR5, showed transitory effects on the heart with minimal cardiac side effects [183]. Administration of RP001 in the SAH model promoted neurological outcomes and cardiac function through inhibition of apoptosis, white matter damage, and microglial/astrocyte activation in the brain [184].

### IL-1Ra

Several studies reported a significant increase in inflammatory responses post-SAH [185]. IL-1 is a critical pro-inflammatory cytokine that mediates brain damage. IL-1 and its downstream molecules, especially IL-6, present significantly deleterious neuropathological states, such as intracerebral hemorrhage [186]. According to previous studies, IL-1 can regulate BBB permeability and exert neurotoxic effects, and its neutralization with monoclonal antibodies reduces vasospasm in experimental aSAH models [187]. Clinical studies have reported that levels of IL-1 and IL-6 are positively associated with the occurrence of cerebral vasospasm following SAH [188, 189]. Additionally, hemoglobin and its decomposition products elicit an IL-1-mediated inflammatory response after a hemorrhagic stroke, which is responsible for the subsequent neurotoxicity [190]. Anakinra, a recombinant form of interleukin-1 receptor antagonist (IL-1Ra), is a natural antagonist to IL-1. Studies on acute neurodegenerative disease and aSAH patients established and demonstrated the biosecurity of anakinra [191-193]. Peripherally administered anakinra has been illustrated to cross the BBB post-SAH and traumatic brain injury [194, 195]. A phase II study also proved that anakinra is safe and well-tolerated and can effectively prevent IL-1-mediated responses following aSAH [196].

### Immunosuppressor minocycline

Minocycline, a specific inhibitor of microglia, has been considered a potential neuroprotective candidate owing to its ability to restrain microglial activation, inhibit T lymphocytic migration, and decrease chemokine and MMP (especially MMP-9) secretion, ultimately stabilizing BBB integrity and protecting against vasospasm and DCI [197]. MMP-9 is known to mediate neutrophil-endothelial interaction and is involved in the breakdown of the BBB following SAH [198, 199]. A previous clinical study reported increased serum MMP-9 levels as a critical mediator for BBB permeability and subsequent cerebral vasospasm [200, 201]. It has been proven that both MMP-9 deletion and administration of minocycline can alleviate SAH-induced vasospasm, improve neurological function, and attenuate cerebral hemorrhage and edema in animal models [200, 202-205]. A small randomized controlled trial (RCT) revealed that minocycline is well-tolerated in aSAH patients, and minocycline administration curtails BBB disruption as evidenced by the reduced permeability post-SAH [206]. However, larger RCTs are required to evaluate the therapeutic potential of minocycline for SAH treatment.

### CAM-targeting therapy

CAMs link the leukocytes and endothelial cells. Specific CAMs, such as ICAM-1 (CD54), integrins (LFA-1 and Mac-1), and selectins, are involved in leukocyte migration into the periadventitial space after hemorrhage. Selectins initiate leukocyte migration and promote the adhesion of leukocytes onto the endothelial surface. Chemo-attractants and the corresponding receptors mediate subsequent events of leukocyte emigration. The interaction between integrins and CAMs mediates the adhesion and trans-endothelial migration of neutrophils to the injury sites [207, 208], which has been reported to be a crucial immunological process post-SAH. ICAM-1 is the receptor for LFA-1 (a heterodimer composed of CD18 and CD11) and is located on the surface of leukocytes or circulating lymphocytes. Several experimental studies revealed elevated levels of ICAM-1 in the cerebral vasculature of animal SAH models [209]. Additionally, selectins and ICAMs were observed in the CSF of patients following SAH [210]. It has also been demonstrated that the administration of anti-ICAM-1 monoclonal antibodies effectively attenuated cerebral vasospasm in rabbit SAH model [84]. Moreover, the efficacy of anti-LFA-1 antibodies has also been confirmed in rabbit chronic post-hemorrhagic vasospasm model. Similar results were reported in the SAH model of cynomolgus monkeys, which confirmed the therapeutic role of the humanized anti-CD11/18 monoclonal antibodies against chronic cerebral vasospasm post-SAH [73]. Moreover, anti-E-selectin antibodies were also reported to be effective in the prevention of SAH-induced vasospasm [211].

**Table 2:** Review of studies using agents targeting the immune system in SAH models.

Drug	Species	Therapeutic window	Dose	SAH model	Observation window	Outcomes	Year	Ref.
Minocycline	Sprague-Dawley rat	1, 2, 3 days before SAH	45; 135mg/kg	Endovascular perforation	24 h, 72h, 21-28 d	High-dose minocycline improved long-term spatial memory and attenuated neuronal loss in the hippocampus and cortex.	2011	Sherchan [237]
Minocycline	Sprague-Dawley rat	0.5 h and 12 h post-SAH	45 mg/kg	Cisterna magna injection	24 h	Minocycline can protect against EBI after SAH.	2011	Guo [238]
Minocycline	Sprague-Dawley rat	1 h post-SAH	135 mg/kg	Endovascular perforation	24 h	Minocycline reduced the cortical levels of ROS and protects against inflammation and apoptosis in EBI.	2016	Li [204]
Minocycline	C57BL/6 mice-based WT/MMP9^-/-^	2 h before SAH, 1 h post-SAH and every 12 h	45 mg/kg	Endovascular perforation	1, 2, 3 d	Minocycline administration to MMP9^-/-^ mice did not yield additional protection.	2017	Vellimana [200]
Minocycline	New Zealand Rabbit	2 h before SAH, 1 h post-SAH and every 12 h	3 mg/kg	Cisterna magna injection	1, 2, 3 d	Both pre- and post-SAH administration of minocycline attenuated SAH-induced vasospasm in rabbits.	2017	Vellimana [200]
Minocycline	Sprague-Dawley rat	2 h and 12 h post-SAH	25g; 50mg/kg	Endovascular perforation	48 h	Treatment with a high dose of minocycline improved the neurological function score, and attenuated cerebral hemorrhage and edema	2019	Li [202]
Minocycline	Sprague-Dawley rat	1, 2, 3 days before SAH	50 mg/kg	Cisterna magna injection	6 h, 7 d	Minocycline administration attenuated the upregulation of TNF-α and IL-1β; inhibited SAH-induced neuronal death and cerebral vasospasm; improved outcomes.	2021	Chung [203]
Minocycline	Sprague-Dawley rat	4 h post-SAH; repeated daily until day 7, every two days until day 14	45 mg/kg	Endovascular perforation	14 d	Minocycline educed phagocytic activity of microglia/macrophages accompanied by a lowered spatial interaction with neurons and reduced neuronal apoptosis inhibit pro-inflammatory cytokines.	2022	Blecharz [205]
Anti-ICAM-1 mAb	Sprague-Dawley rat	3 h, 3, 6, and 9 d post-SAH	2 mg/kg	Rat femoral artery model	24 h, 12 d	Systemic administration anti-ICAM-1 mAb chronic inhibits vasospasm and is correlated with a reduction in the infiltration of macrophages and granulocytes in the periadventitial region of blood-exposed arteries	1997	Oshiro [185]
Anti-ICAM-1; Anti-CD18	New Zealand Rabbit	Intracisternal injection before SAH	10 mg	Cisterna magna injection	2 d	Severity of cerebral vasospasm can be attenuated using monoclonal antibodies against ICAM-1 and CD18.	1998	Bavbek [84]
Anti-LFA-1 mAb; anti-ICAM-1 mAb	Sprague-Dawley rat	3 h, 3, 6, and 9 d post-SAH	2 mg/kg	Rat femoral artery model	24 h, 12 d	Systemic administration of anti-LFA-1 or anti-ICAM-1 mAb inhibits the development of delayed chronic vasospasm and leads to a significant reduction in periadventitial inflammatory cells.	2002	Clatterbuck [239]
Humanized anti-CD11/CD18 mAb	Cynomolgus monkeys	0.5 to 1 h post-SAH	2 mg/kg	Cisterna magna injection	7 d	Humanized anti-CD11/18 mAb is feasible in the treatment of chronic cerebral vasospasm after experimental SAH.	2003	Clatterbuck [73]
Humanized anti-CD11/CD18 mAb Hu23F2G	New Zealand rabbits	0.5, 24, and 48 h post-SAH	4 mg/kg	Cisterna magna injection	3 d	Anti-CD11/CD18 mAb prevents vasospasm after SAH by inhibiting adhesion of neutrophils and macrophages and their migration into the periadventitial space.	2004	Pradilla [211]
Anti-E selection	C57BL/6 mice	Immediately post-SAH	12.5, 4, 1 mg	Cisterna magna injection	24 h	Anti-E-selectin antibody was effective in prevention of SAH-induced vasospasm	2005	Lin [240]
FTY720 Fingolimod	Sprague-Dawley rat	3 h post SAH	0.5 mg/kg	Endovascular perforation	2 d, 7 d	Treatment with fingolimod reduced the intravascular leukocyte adhesion to pial venules, preserved pial arteriolar dilating function, and improved neurological outcome in rats subjected to SAH.	2015	Xu [182]
FTY720 Fingolimod	Sprague-Dawley rat	0.5 h post SAH	1mg/kg	Endovascular perforation	24 h, 72 h	Systemic administration of FTY720 ameliorated SAH-induced neurological deficits and brain edema without modulation of CBF and the amount of subarachnoid blood.	2017	Hasegawa [180]
FTY720 Fingolimod	Sprague-Dawley rat	i.c.v 1.5 h before SAH	1 μg	Endovascular perforation	24 h	FTY720 induced PP2A activation would lead to dephosphorylation and activation of TTP and decreased production of TNF-α, IL-6, and IL-8.	2018	Yin [179]
RP001	C57BL/6 mice	i.p. 0.5 h, 24 h, 48 h post SAH	0.3; 0.5 mg/kg	Endovascular perforation	3 d	Low-dose-RP001 treatment decreases apoptosis, white matter damage, blood brain barrier permeability, microglial/astrocyte activation, MCP-1, MMP-9 and NADPH oxidase-2 expression in the brain.	2019	Li [184]
FTY720 Fingolimod	C57BL/6 mice	i.p. immediately post SAH	1 mg/kg	Endovascular perforation	1-7 d, 10 d, 14 d	Fingolimod increased Treg cells and down-regulated NK cells and inhibited the expression of inflammatory cytokines in brain tissue.	2020	Wang [181]
Human-IL-1Ra Kineret (Anakinra)	Wistar rats	Sc. injection 0.5 h after SAH	75 mg/kg	Endovascular perforation	2 d	BBB breakdown is correlated with brain damage and is reduced by IL-1Ra	2012	Greenhalgh [190]
Anti-HMGB1 mAb	Wistar rats	i.v. injection twice with a 24 h interval post SAH	1 mg/kg	Endovascular perforation	2 d	Anti-HMGB1 mAb attenuated the enhanced vasocontractile response to thrombin of the isolated BA and prevented activation of microglia; locomotor activity and weight loss recovery were enhanced.	2016	Haruma [222]
Anti-HMGB1 mAb	Sprague-Dawley rats	i.v. administered twice with a 24 h interval post SAH	1 mg/kg	Endovascular perforation	3 d	Anti-HMGB1 mAb reversed VSMC phenotypic switching and vascular remodeling in rats; relieved ischemia; reduced neurological impairment; ameliorated the increased microglial activation	2019	Wang L [127]
Anti-mouse/rat TNF-α	Wistar rats	Microinfused 0.5 h before SAH	250 ng/lL, 10 lL	Cisterna magna injection	24 h; 48h	Anti-TNF-a antibody blocked apoptosis induced by SAH in the hippocampus.	2012	Jiang [241]
Adalimumab	New Zealand Rabbit	i.p injection once a day for 3 d.	5 mg/kg/day	Cisterna magna injection	72 h	ADA treatment increased the mean cross-sectional area of the vasospastic BA, reduced the BA wall thickness; ameliorated enhanced endothelial apoptosis	2020	Toğuşlu [229]

### Circulatory factor-targeting therapy

#### Anti-complement antibodies

Several studies reported that the complement cascade is activated following SAH and that it is associated with poor prognosis of SAH patients [212]. The pro-inflammatory anaphylatoxins, such as C3a and C5a, are responsible for vasoconstriction, activated coagulation, and aggravated EBI and DCI [213, 214]. C5a levels were significantly increased in SAH patients compared to those with unruptured intracranial aneurysms [36]. It has also been revealed that C5-targeting antibodies prevent the formation of C5a, which can further inhibit microglial activation and cell death in mouse SAH model [36]. Notably, eculizumab, a C5 antibody, is used for the management of several inflammatory diseases, including neuromyelitis optica and myasthenia gravis [215, 216]; however, its therapeutic effects in SAH have not yet been revealed. A phase II trial has been conducted to investigate the pharmacodynamic efficacy and biosecurity of eculizumab in aSAH patients in the acute phase [217].

#### Anti-HMGB1 antibodies

Increased intracranial pressure induces the release of DAMPs from damaged cells [18]. DAMP receptors are localized on several cells, including ECs, neurons, and immune cells. Inflammatory responses may be initiated in the brain parenchyma and cerebral vessel through binding to TLRs, such as TLR-2 and TLR-4 [218]. HMGB1 is a DAMP released from damaged cells, which binds to PRRs located on immune cells and mediates inflammatory responses [219, 220]. The release of HMGB1 can be observed in the CSF of SAH patients and elevated levels of HMGB1 indicate poor prognosis [221]. Studies have revealed that HMGB1 can be considered a potential target to attenuate brain damage and alleviate functional outcomes post-SAH. The administration of anti-HMGB1 antibodies in a rat SAH model effectively attenuated vasocontractile response, prevented activation of cerebrocortical microglia, and enhanced locomotor activity and weight loss recovery [222]. Furthermore, another study demonstrated that the administration of anti-HMGB1 antibodies improved blood flow, reduced brain edema, and inhibited inflammatory activation and neurological impairments [127].

#### Anti-TNF-α antibodies

TNF-α, another critical proinflammatory cytokine, is released by activated macrophages and astrocytes [223-225]. It mediates systemic inflammation and plays a critical role in the progression of CV post-SAH by accelerating the inflammatory response cascade [226]. In addition, TNFα and IL-1β also play essential roles in the activation of the apoptotic process [227]. In a previous study, immuno-reactivities of TNF-α in the basilar artery and CSF post-SAH were inhibited by a p38 mitogen-activated protein kinase (MAPK) inhibitor, SB203580. Moreover, SB203580 is considered a potential therapeutic agent against cerebral vasospasm post-SAH [228]. Adalimumab (ADA), a recombinant human IgG1 monoclonal antibody targeting TNF-α, prevents the binding between TNF-α and its receptor [191]. According to a series of experimental studies, administration of ADA markedly enlarges the mean cross-sectional area of the vasospastic basilar arteries (BA), decreases the arterial wall thickness, and ameliorates endothelial apoptosis [229].

#### Anti-CCL20 antibodies

CCL20 is located on epithelial and various immune cells under normal and homeostatic conditions, while it is upregulated under inflammatory conditions and mediates inflammatory cascade reactions [230-233]. A recent study reported that CCL20 is upregulated after SAH [234], and it was confirmed that CCL20 is predominantly located on both neurons and microglia and that it triggers inflammatory responses and neuronal apoptosis via enhanced CCR6 activity post-SAH. Therefore, the administration of CCL20-neutralizing antibodies can attenuate neurological deficits, alleviate brain edema, and inhibit neuronal apoptosis post-SAH [235].

## Conclusions and future directions

Several *in vivo* and *in vitro* studies have provided us with insight into the interactions between systemic immunity and the CNS post-SAH, which will help in harnessing the therapeutic effects of immune responses for SAH treatment. Our review highlights the essential function of innate and adaptive immunity on the clinical and functional outcomes of SAH patients, and with the continuously improving understanding of the immunological mechanisms, we obtain a more detailed understanding of the diverse roles of innate and adaptive immunity in the progression of SAH. In this review, we discussed the dual role of the immune system, including the pro-inflammatory responses and immunosuppressive effects post-SAH onset. Furthermore, we summarized the experimental and clinical studies of various immunotherapies used in SAH treatment. Immunotherapy can be used as a complementary treatment to conventional treatments to improve the prognosis and minimize side effects; however, in some cases, immunotherapy may cause systemic side effects or harm organ systems. Therefore, therapeutic strategies for SAH treatment must be tailored to the particular stage of SAH and the associated inflammatory responses.
